# Stress-Activated Protein Kinase OsSAPK9 Regulates Tolerance to Salt Stress and Resistance to Bacterial Blight in Rice

**DOI:** 10.1186/s12284-019-0338-2

**Published:** 2019-11-11

**Authors:** Fan Zhang, Dan Zeng, Liyu Huang, Yingyao Shi, Tengjun Chen, Fan Zhang, Yongli Zhou

**Affiliations:** 1 Institute of Crop Sciences/National Key Facility for Crop Gene Resources and Genetic Improvement, Chinese Academy of Agricultural Sciences, 12 South Zhong-Guan-Cun Street, Beijing, 100081 China; 2 Graduate School of Chinese Academy of Agricultural Sciences, 12 South Zhong-Guan-Cun Street, Beijing, 100081 China; 3 School of Agriculture, Yunnan University, Kunming, China; 4 College of Agronomy, Anhui Agricultural University, Hefei, China

**Keywords:** Rice, The SnRK2 family, *OsSAPK9*, Salt stress, Bacterial blight, OsSTG1

## Abstract

**Background:**

Salt stress and bacterial blight caused by *Xanthomonas oryzae* pv. *oryzae* (*Xoo*) are key limiting factors of rice (*Oryza sativa* L.) yields. Members of sucrose non-fermenting 1 (SNF1)-related protein kinase 2 (SnRK2), which is a family of plant-specific Ser/Thr kinases, are important components of signaling pathways involved in plant developmental processes and responses to stresses. There are 10 members of the SnRK2 family in rice; however, their functions are poorly understood, as are the underlying molecular mechanisms.

**Results:**

In this study, we found that *OsSAPK9,* which belongs to the SnRK2 family, positively regulated salt-stress tolerance and strain-specific resistance to bacterial blight in rice. RNA sequencing revealed that there were 404 and 1324 genes differentially expressed in *OsSAPK9*-*RNAi* in comparison with wild-type plants under salt-stress conditions and after *Xoo* inoculation, respectively, which participate in basic metabolic processes. In total, 65 common differentially expressed genes involved mainly in defense responses were detected both under salt-stress conditions and after *Xoo* inoculation. Moreover, in vivo and in vitro experiments demonstrated that OsSAPK9 forms a protein complex with the molecular chaperones OsSGT1 and OsHsp90, and transgenic plants overexpressing *OsSGT1* exhibited decreased tolerances to salt stress and significantly increased resistance levels to bacterial blight. Thus, OsSAPK9 may function as a center node regulator of salt-stress responses and disease-resistance pathways through its interaction with OsSGT1 in rice.

**Conclusion:**

This study confirms that OsSAPK9 functions as a positive regulator of salt-stress responses and disease resistance through its interaction with OsSGT1 in rice.

## Background

Rice (*Oryza sativa* L.) is an important staple food crop for more than half the global population. The large worldwide area for rice cultivation has led to its growth in diverse ecosystems in which it is exposed to diverse stresses. Soil salinization and bacterial blight caused by *Xanthomonas oryzae* pv. *oryzae* (*Xoo*) are two of the main constraints that lead to significant yield losses in rice growing regions in China and South/Southeast Asia (Nino-Liu et al. 2006). Plants have developed sophisticated mechanisms to respond to environmental stresses (Fujita et al. 2006; Sharma et al. 2013). Identifying the key components in the signaling pathways involved in these stress responses will provide information needed to breed tolerance to multiple stresses and improve rice yields. Members of sucrose non-fermenting 1 (SNF1)-related protein kinase 2 (SnRK2), which is a family of plant-specific Ser/Thr kinases, are important components of the signaling pathways involved in abscisic acid (ABA)-dependent developmental processes and responses to abiotic stresses (Fujii et al. 2011; Kulik et al. 2011; Yan et al. 2017). There are 10 known *SnRK2* genes in *Arabidopsis* and 11, 8, and 20 known *SnRK2* genes in maize, potato, and cotton, respectively (Bai et al. 2017; Huai et al. 2008; Liu et al. 2017; Saha et al. 2014). The functions of *SnRK2*s in *Arabidopsis thaliana* have been widely studied. *AtSnRK2*s not only function in several developmental processes and responses to saline and drought (Cheng et al. 2017; Grondin et al. 2015; Kim et al. 2012; McLoughlin et al. 2012; Soma et al. 2017; Tan et al. 2018; Yoshida et al. 2002; Zheng et al. 2010), but they are also involved in disease resistance. For example, *AtSnRK2.8* mediates phosphorylation and salicylic acid signals, which coordinately function to activate NPR1 through a dual-step process that leads to systemic immunity (Lee et al. 2015). At present, 10 members of the SnRK2 family have been identified in rice and are designated stress-activated protein kinases1–10 (*OsSAPK1*–*10*) (Kobayashi et al. 2004). The expression levels of all 10 *OsSAPK*s are up-regulated under salt-stress conditions. The overexpression of *OsSAPK4* increases the tolerance to oxidative stresses (Diédhiou et al. 2008), while the overexpression *SAPK9* from *Oryza rufipogon* in a drought-sensitive rice line enhances drought tolerance and yield-related traits (Dey et al. 2016). Conversely, *sapk2* mutants are more sensitive to drought stress than wild-type (WT) plants (Lou et al. 2017). *OsSAPK3*, *OsSAPK5*, *OsSAPK7*, and *OsSAPK9* are up-regulated when the transgenic rice line carrying the heterologous resistance gene *Rxo1* is inoculated with *Xanthomonas oryzae* pv. *oryzicola* (Xu et al. 2013), while *OsSAPK2* knock-down mutants increase the susceptibility to bacterial blight (Hu et al. 2015). However, while *OsSAPK*s might be associated with responses to abiotic and biotic stresses in rice, the functions of this gene family are poorly understood and their underlying molecular mechanisms have yet to be elucidated. In the present study, we used *OsSAPK9-RNAi* and *OsSAPK9-overexpression* transgenic lines to show that *OsSAPK9* is involved in tolerance to salt stress and resistance to bacterial blight. We also showed that OsSAPK9 interacts with OsSGT1 to regulate these processes. Additionally, we used transcriptome profiling to investigate the defense responses to salt stress and *Xoo* infection mediated by *OsSAPK9*.

## Results

### *OsSAPK9* Expression in Rice in Response to Salt Stress and *Xoo* Infection

Each of the 10 members of the rice SnRK2 family, including *OsSAPK9*, are activated by hyperosmotic stress in cultured cell protoplasts (Kobayashi et al. 2004). Here, we demonstrated that *OsSAPK9*’s expression was rapidly induced 2 h after rice seedlings were treated with 100 mM NaCl (Additional file 1: Figure S1a). In addition, *OsSAPK9*’s transcription levels in rice plants increased more than 3-fold at 6 h and 72 h after inoculation with the *Xoo* strain GD1358 (Additional file 1: Figure S1b). Thus, *OsSAPK9* may be up-regulated in response to salt stress and *Xoo* infection.

### *OsSAPK9* Positively Regulates Tolerance to Salt Stress in Rice

**Fig. 1:**
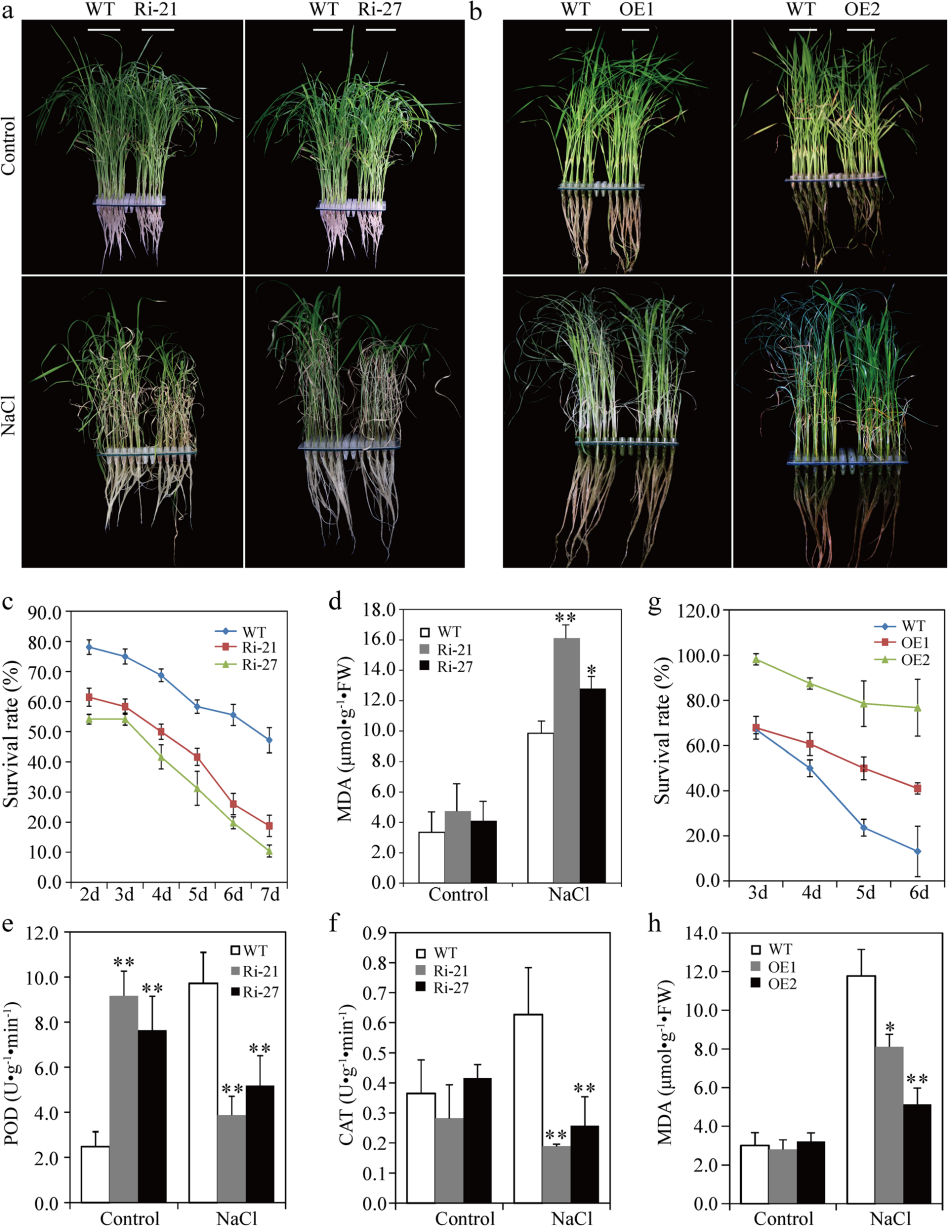
Phenotypic responses of *OsSAPK9-RNAi*, *OsSAPK9-overexpression* (*OsSAPK9-OE*), and wild-type (WT) plants to salt-stress conditions. **a** Phenotypes of *OsSAPK9-RNAi* and WT plants treated with or without 100 mM NaCl. Photographs were taken 6 d after treatment. **b** Phenotypes of *OsSAPK9-OE* and WT plants treated with or without 100 mM NaCl. Photographs were taken 6 d after treatment. **c** Survival rates of *OsSAPK9-RNAi* and WT plants under salt-stress conditions (100 mM NaCl). **d** Malondialdehyde (MDA) contents in *OsSAPK9-RNAi* and WT plants at 6 d after being treated with 100 mM NaCl. **e** Peroxidase (POD) and **(f)** Catalase (CAT) activities in *OsSAPK9-RNAi* and WT plants 6 d after being treated with 100 mM NaCl. **g** Survival rates of *OsSAPK9-OE* and WT plants under salt-stress conditions (100 mM NaCl). **h** MDA contents in *OsSAPK9-OE* and WT plants 6 d after being treated with 100 mM NaCl. Values are presented as the means and standard errors of three replicates. *P < 0.05, according to a Student’s *t*-test

To determine the biological function of *OsSAPK9*, *OsSAPK9-RNAi* (denoted with Ri) and *OsSAPK9-overexpression* (denoted with OE) transgenic rice lines were generated (Additional file 2: Figure S2). The phenotypic responses of *OsSAPK9*-*RNAi*, *OsSAPK9*-*OE*, and WT plants to salt stress were examined. *OsSAPK9*-*RNAi* lines Ri-21 and Ri-27 were more sensitive to salt stress in comparison with the WT, and the survival rates of Ri-21 and Ri-27 were significantly reduced 7 d after treatment with 100 mM NaCl (Fig. 1a, c). *OsSAPK9*-*OE* lines OE1 and OE2 were more tolerant to the salt treatment, and their survival rates significantly increased in comparison with the WT (Fig. 1b, g). To examine the physiological changes in salt-stressed *OsSAPK9*-*RNAi* and *OsSAPK9*-*OE* lines, we measured known physiological parameters that are associated with salt stress. The accumulation of malondialdehyde (MDA) increased considerably under salt-stress conditions in shoots of *OsSAPK9*-*RNAi*, *OsSAPK9*-*OE*, and WT plants compared with under normal conditions (Fig. 1d, h). When plants were treated with NaCl, the MDA contents were significantly higher in the *OsSAPK9*-*RNAi* than in the WT plants and significantly lower in *OsSAPK9*-*OE* plants than in the WT plants (Fig. 1d, h). After treatment with 100 mM NaCl, the peroxidase (POD) and catalase (CAT) activities in the *OsSAPK9*-*RNAi* plants were significantly lower than those in the WT plants (Fig. 1e, f). Thus, *OsSAPK9* positively regulates rice tolerance to salt stress. The shoots of *OsSAPK9*-*RNAi* lines Ri-21 and Ri-27 were shorter than those of the WT plants after treatments with 50 μm NaCl; however, there were no significantly differences in the root lengths of these plants (Additional file 3: Figure S3a, b). The shoot dry weights and root dry weights of the Ri-21 and Ri-27 lines were significantly less than those of the WT plants (Additional file 3: Figure S3c). Thus, the suppression of *OsSAPK9* appears to significantly decrease salt tolerance in rice.

### *OsSAPK9* Increases Strain-Specific Resistance to Bacterial Blight in Rice

**Fig. 2:**
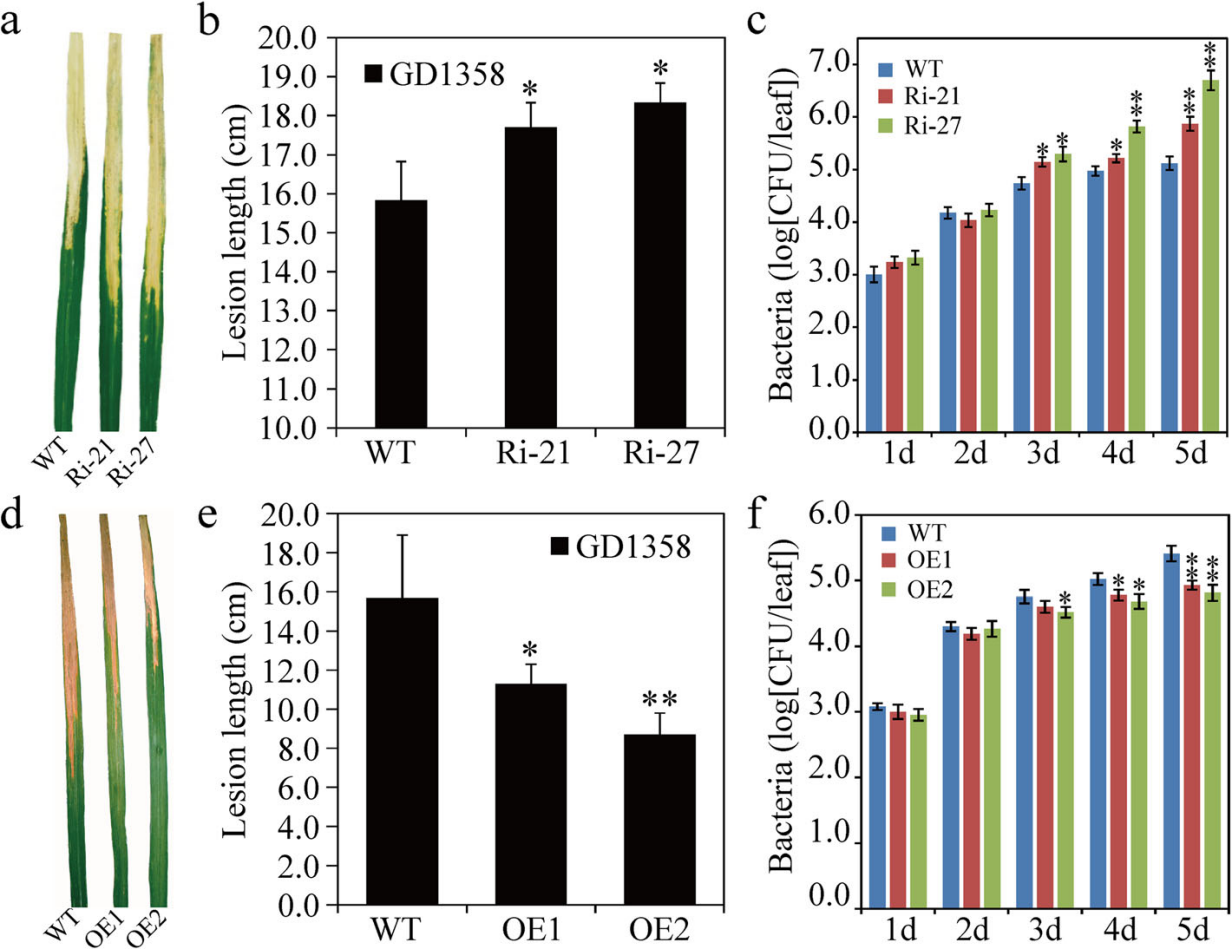
Phenotypic responses of *OsSAPK9-RNAi*, *OsSAPK9*-*overexpression* (*OsSAPK9-OE*), and wild-type (WT) plants to infection by *Xanthomonas oryzae* pv. *oryzae* (*Xoo*) strain GD1358. **a** Phenotypes of *OsSAPK9-RNAi* and WT plants infected with *Xoo* strain GD1358. Photographs were taken 12 d after inoculation. **b** Lesion lengths of bacterial blight in the *OsSAPK9-RNAi* and WT plants 12 d after inoculation with *Xoo* strain GD1358. **c** Growth curves of the *Xoo* strain GD1358 in *OsSAPK9-RNAi* and WT plants. CFU, colony-forming units. **d** Phenotypes of *OsSAPK9-OE* and WT plants infected with *Xoo* strain GD1358. Photographs were taken 12 d after inoculation. **e** Lesion lengths of bacterial blight in the *OsSAPK9-OE* and WT plants 12 d after inoculation with *Xoo* strain GD1358. **f** Growth curves of *Xoo* strain GD1358 in *OsSAPK9-OE* and WT plants. CFU, colony-forming units. Values are presented as the means and standard errors of three replicates. *P < 0.05, according to a Student’s *t*-test

To investigate whether *OsSAPK9* confers tolerance to bacterial blight in rice, *OsSAPK9*-*RNAi*, *OsSAPK9*-*OE*, and WT plants were separately inoculated with four *Xoo* strains (GD1358, JS97–2, PXO340, and PXO347). When the *OsSAPK9*-*RNAi* transgenic lines were inoculated with GD1358, the lesion lengths (LLs) were significantly longer than those of the WT plants and the bacterial populations were significantly increased (Fig. 2a–c). In contrast, when the *OsSAPK9*-*OE* transgenic lines were inoculated with GD1358, the LLs were significantly shorter than those of the WT plants and the bacterial growth was reduced (Fig. 2d–f). *OsSAPK9*-*RNAi* and *OsSAPK9*-*OE* transgenic lines inoculated with PXO347 showed similar responses to those observed following GD1358 inoculation (Additional file 4: Figure S4a, b, g, h). However, no significant differences were detected between the LLs of both the *OsSAPK9*-*RNAi* and *OsSAPK9*-*OE* transgenic lines and the WT plants when *Xoo* strains PXO340 and JS97–2 were tested (Additional file 4: Figure S4c–f, i–l). Thus, *OsSAPK9*’s overexpression may positively regulate strain-specific resistance to bacterial blight in rice.

### Interaction Between OsSAPK9 and the Molecular Chaperone Protein OsSGT1

**Fig. 3:**
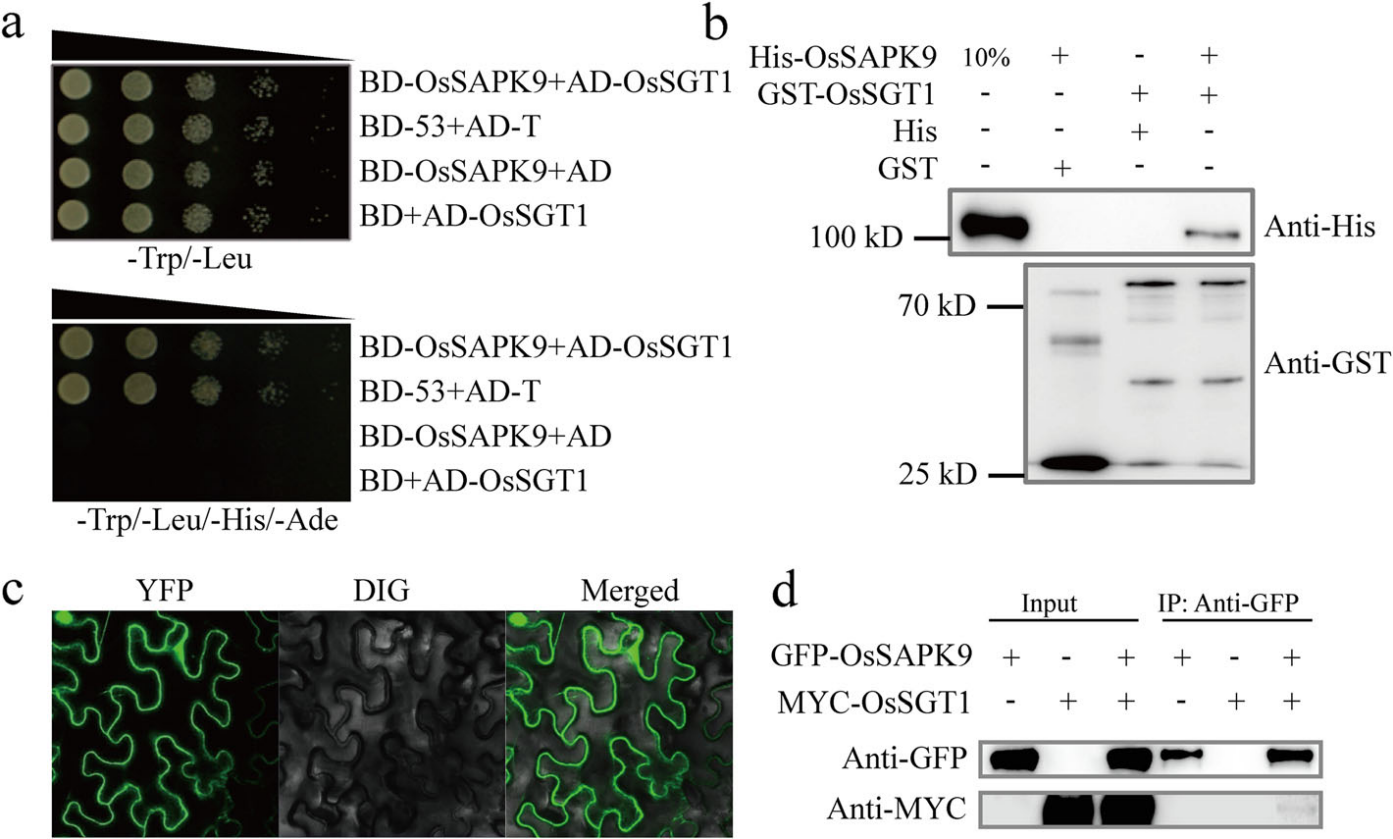
OsSAPK9 interactions with OsSGT1 in vitro and in vivo. **a** OsSAPK9 interaction with OsSGT1 in yeast. AH109 yeast transformants diluted 10, 100, 1000, or 10,000 times were plated on synthetic dextrose (SD) medium lacking Trp and Leu amino acids (SD − L/T) or SD medium lacking Trp, Leu, His, and Ade amino acids (SD − L/T/W/A). **b** OsSAPK9 bound to OsSGT1 in the glutathione S-transferase (GST) pull-down assay. **c** OsSAPK9 interaction with OsSGT1 in *Nicotiana benthamiana* cells in the bimolecular fluorescence complementation assay. OsSAPK9 was fused to an N-terminal yellow fluorescent protein (NYFP-OsSAPK9) and OsSGT1 was fused to a C-terminal cyan fluorescent protein (CCFP-OsSGT1). **d** OsSAPK9 bound to OsSGT1 in the co-immunoprecipitation assay. Constructs encoding green fluorescent protein-tagged OsSAPK9 and Myc-tagged OsSGT1 were co-expressed in *Agrobacterium*-infiltrated *N. benthamiana* cells. Tissues were harvested 3 d after infiltration

**Fig. 4:**
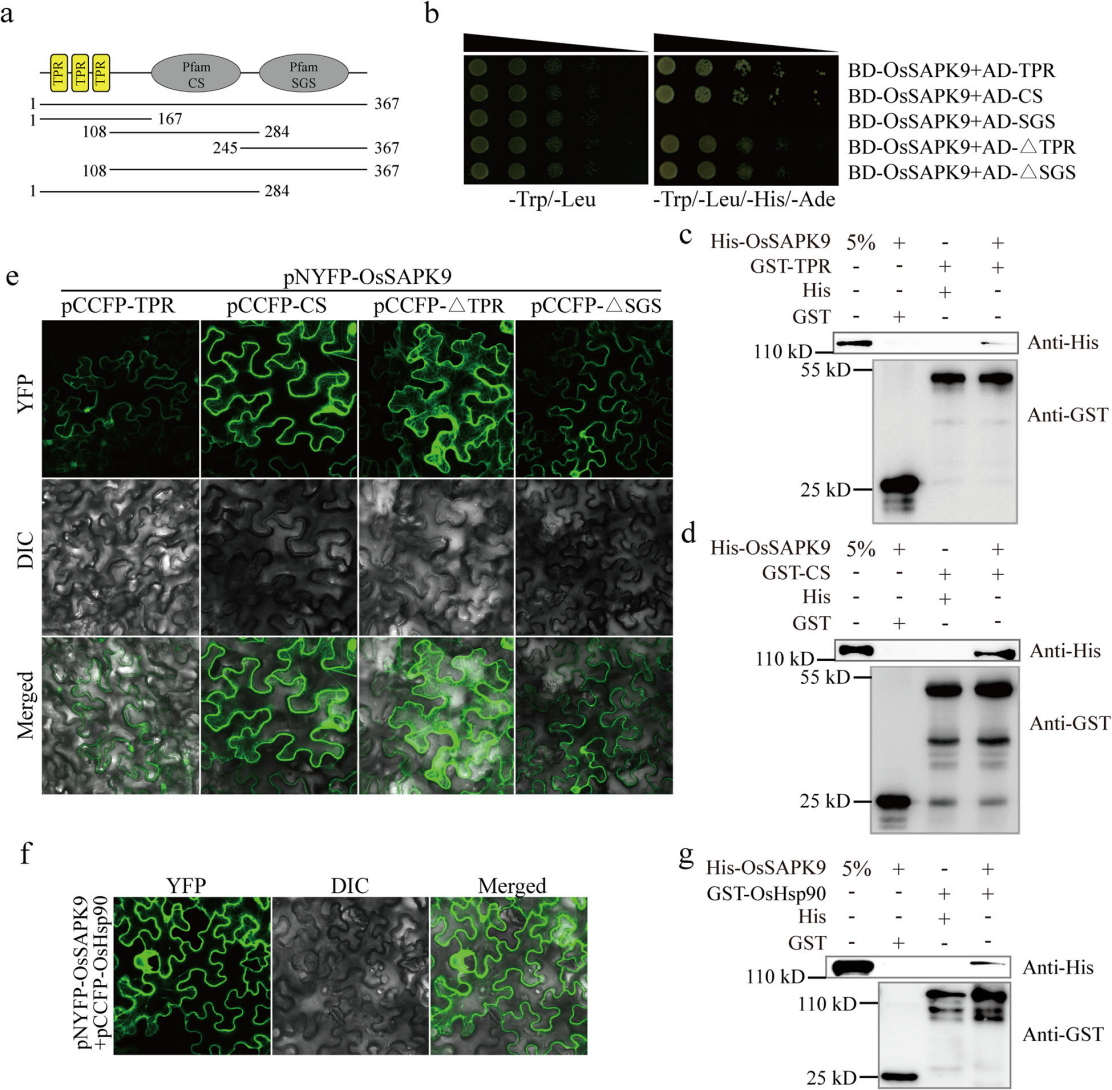
OsSAPK9 interactions with different OsSGT1 domains in vitro and in vivo. **a** Schematic of the functional domain of OsSGT1. Positions of the tetratricopeptide repeat (TPR) domain (1–167 amino acids), the CHORD-containing protein and SGT1 (CS) domain (108–284 amino acids), the SGT-specific (SGS) domain (245–367 amino acids), the ΔTPR domain (108–367 amino acids), and the ΔSGS domain (1–284 amino acids) are indicated. **b** OsSAPK9 interactions with the TPR and CS domains in yeast. The TPR, CS, SGS, ΔTPR, and ΔSGS domains are from OsSGT1. AH109 yeast transformants diluted 10, 100, 1000, or 10,000 times were plated on synthetic dextrose (SD) medium lacking Trp and Leu amino acids (SD − L/T) or SD medium lacking Trp, Leu, His and Ade amino acids (SD − L/T/W/A). **c** OsSAPK9 bound to the TPR domain of OsSGT1 in the glutathione S-transferase (GST) pull-down assay involving His-OsSAPK9 and GST-TPR. **d** OsSAPK9 bound to the CS domains of OsSGT1 in the glutathione S-transferase (GST) pull-down assay involving His-OsSAPK9 and GST-CS. **e** OsSAPK9 interactions with the TPR and CS domains in a bimolecular fluorescence complementation assay. OsSAPK9 was fused to an N-terminal yellow fluorescent protein (NYFP-OsSAPK9), and TPR, CS, SGS, ΔTPR, and ΔSGS were fused to C-terminal cyan fluorescent proteins (CCFP-TPR, CCFP-CS, CCFP-SGS, CCFP-ΔTPR, and CCFP-ΔSGS). **f** OsSAPK9 interactions with OsHsp90 in the bimolecular fluorescence complementation assay. OsSAPK9 was fused to an N-terminal yellow fluorescent protein (NYFP-OsSAPK9), and OsHsp90 was fused to a C-terminal cyan fluorescent protein (CCFP-Hsp90). **g** OsSAPK9 bound to OsHsp90 in the GST pull-down assay involving His-OsSAPK9 and GST-OsHsp90

To identify any proteins that interact with OsSAPK9, the *OsSAPK9* protein-coding sequence (CDS) was used as bait to screen a rice-*Xoo* cDNA library using a yeast two-hybrid system. We identified 31 potential interacting molecules (Additional file 8: Table S1), including *OsSGT1*, which encodes a protein involved in disease resistance (Austin et al. 2002; Azevedo et al. 2002). To verify the interaction between OsSAPK9 and OsSGT1, we conducted a yeast two-hybrid, glutathione S-transferase (GST) pull-down, bimolecular fluorescence complementation (BiFC), and co-immunoprecipitation (Co-IP) assays. Yeast cells co-transformed with the constructs encoding AD-OsSGT1 and BD-OsSAPK9, or BD-53 and AD-T (the positive controls), were able to grow in synthetic dextrose (SD)^4−^ dropout medium (lacking Leu, Trp, His, and Ade). In contrast, yeast cells co-expressing the constructs encoding AD and BD-OsSAPK9, or BD and AD-OsSGT1 (the negative controls), were unable to grow in the SD^4−^ drop-out medium (Fig. 3a). When the His-OsSAPK9 fusion protein and the His protein control were incubated with recombinant GST-OsSGT1 and GST, respectively, only His-OsSAPK9 was able to bind GST-OsSGT1, indicating that there is a direct interaction between OsSAPK9 and OsSGT1 (Fig. 3b; Additional file 5: Figure S5a). To determine whether OsSAPK9 interacts with OsSGT1 in vivo, we conducted BiFC assays using *Nicotiana benthamiana* plants. In leaves co-expressing *pNYFP-OsSAPK9* and *pCCFP-OsSGT1*, BiFC signals were detected in the cytoplasm and nucleus (Fig. 3c). Co-IP assays using *N. benthamiana* plants expressing green fluorescent protein (GFP)-OsSAPK9 and/or MYC-OsSGT1 revealed that the anti-GFP antibodies pulled down MYC-OsSGT1. The signals were only detectable in the GFP-OsSAPK9 and MYC-OsSGT1 co-expression leaves (Fig. 3d), confirming the interaction between OsSAPK9 and OsSGT1. The tetratricopeptide repeat (TPR), CHORD-containing protein and SGT1 (CS), and SGT-specific (SGS) domains form three distinct regions of SGT1 (Azevedo et al. 2002; Shirasu 2009). The yeast two-hybrid analysis revealed that OsSAPK9 interacted with the TPR, central CS, deleted TPR domain (ΔTPR), and deleted SGS domain (ΔSGS) regions of OsSGT1, while there was no interaction between the C-terminal SGS region of OsSGT1 and OsSAPK9 (Fig. 4a, b). Consistent with these observations, the pull-down and BiFC assays confirmed that OsSAPK9 interacted with the TPR and CS domains of OsSGT1 (Fig. 4c-e; Additional file 5: Figure S5b, c). SGT1 interacts with Hsp90 to promote protein folding and stability in plants (Meldau et al. 2011). In this study, BiFC signals in the cytoplasm and the nucleus were detected in *N. benthamiana* plants co-transformed with the *pNYFP-OsSAPK9* and *pCCFP-OsHsp90* vectors (Fig. 4f). Additionally, we found that GST-OsHsp90 bound to His-OsSAPK9 in pull-down assays, while neither GST nor His bound to His-OsSAPK9 or GST-OsHsp90 (Fig. 4g; Additional file 5: Figure S5d). These results suggest that OsSAPK9, OsSGT1, and OsHsp90 together form a protein complex.

### *OsSGT1* Regulates Rice Responses to Salt Stress and Bacterial Blight

**Fig. 5:**
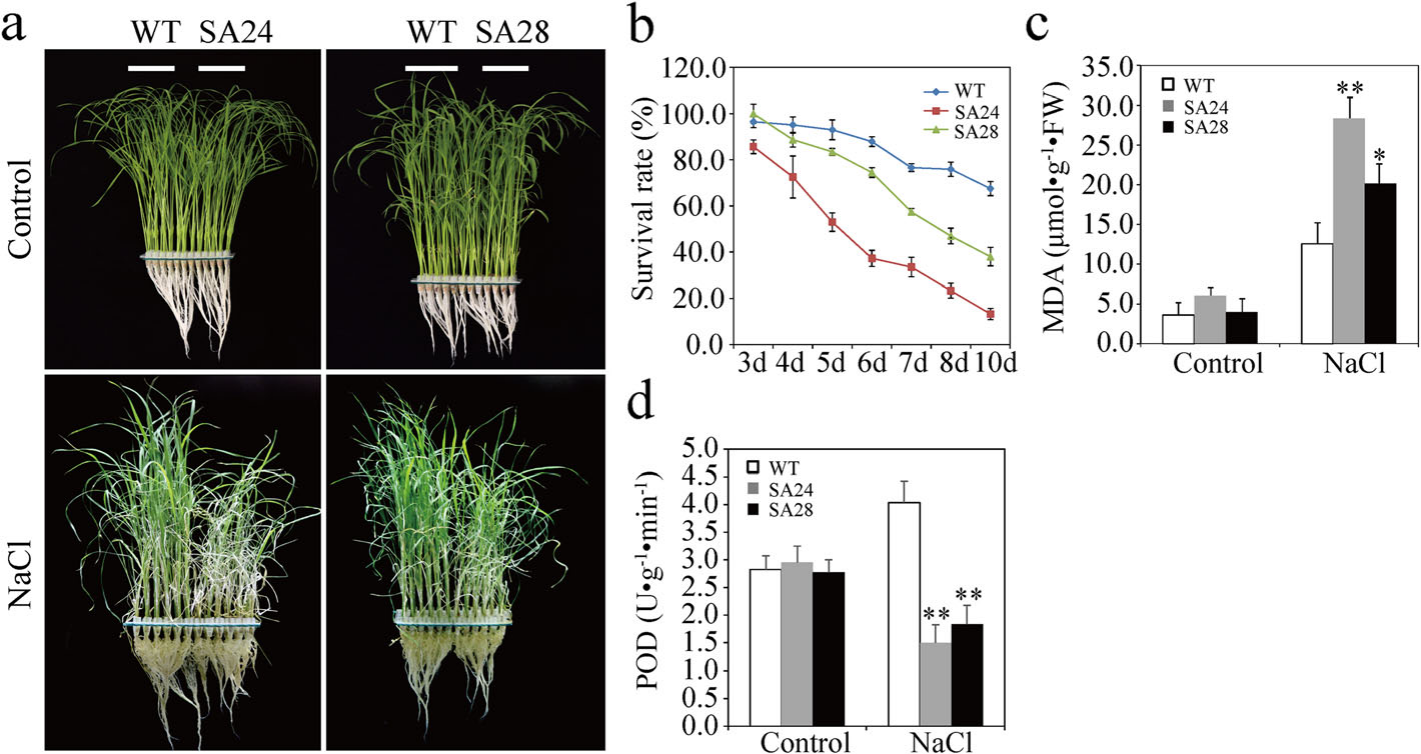
Phenotypic responses of *OsSGT1-overexpression* (*OsSGT1-OE*) and wild-type (WT) plants under salt-stress conditions. **a** Phenotypes of *OsSGT1-OE* and WT plants treated with or without 100 mM NaCl. Photographs were taken 6 d after treatment. **b** Survival rates of *OsSGT1-OE* and WT plants under salt-stress conditions (100 mM NaCl). **c** Malondialdehyde (MDA) contents in *OsSGT1-OE* and WT plants 6 d after being treated with 100 mM NaCl. **d** Peroxidase (POD) activities in *OsSGT1-OE* and WT plants 6 d after being treated with 100 mM NaCl. Values are presented as the means and standard errors of three replicates. *P < 0.05, according to a Student’s *t*-test

**Fig. 6:**
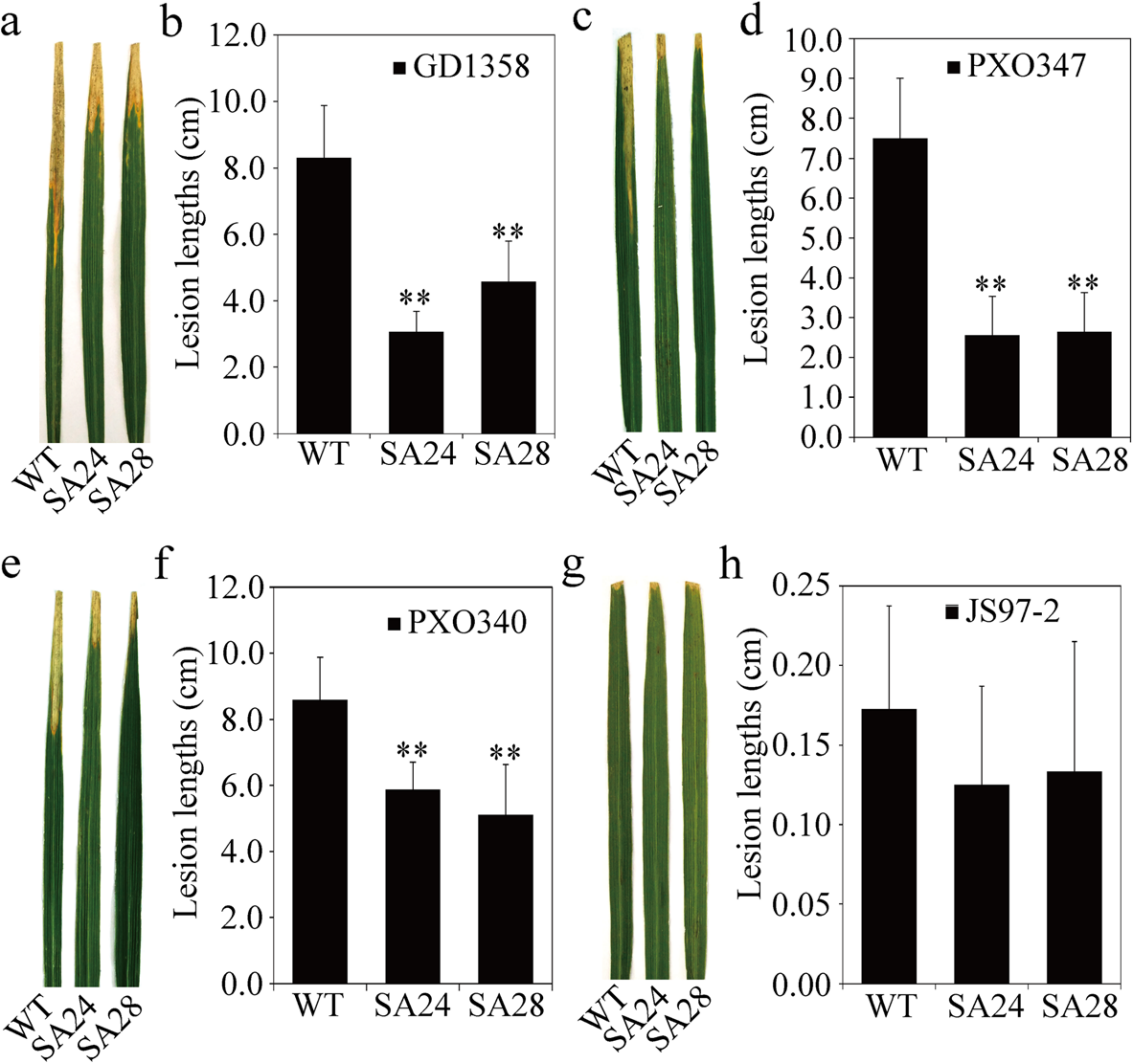
Phenotypic responses of *OsSGT1*-*overexpression* (*OsSGT1-OE*) and wild-type (WT) plants inoculated with *Xanthomonas oryzae* pv. *oryzae* (*Xoo*). **a** and **(b)** Phenotypes of *OsSGT1-OE* and WT plants, respectively, inoculated with *Xoo* strain GD1358. **c** and **(d)** Phenotypes of *OsSGT1-OE* and WT plants, respectively, inoculated with *Xoo* strain PXO347. **e** and **(f)** Phenotypes of *OsSGT1-OE* and WT plants, respectively, inoculated with *Xoo* strain PXO340. **g** and **(h)** Phenotypes of *OsSGT1-OE* and WT plants, respectively, inoculated with *Xoo* strain JS97–2. Photographs were taken 12 d after inoculation. The lesion lengths were measured 12 d after inoculation with *Xoo* strains. Values are presented as the means and standard errors of three replicates. *P < 0.05, according to a Student’s *t*-test

To functionally characterize the interaction between OsSAPK9 and OsSGT1, the responses of *OsSGT1-overexpression* (denoted with SA) transgenic rice lines (SA24 and SA28) to salt stress and *Xoo* inoculation were investigated (Additional file 6: Figure S6). After 10 d of treatment with 100 mM NaCl, the survival rates of the SA24 and SA28 lines were 13.3% and 38.0%, respectively. These rates were significantly lower than that of the WT plants (67.5%; Fig. 5a, b). Furthermore, the MDA contents were significantly greater in *OsSGT1*-*OE* lines than in the WT plants, while the POD activities were significantly weaker (Fig. 5c, d). Thus, *OsSGT1* may negatively regulate rice responses to salt stress. The LLs in the *OsSGT1*-*OE* transgenic rice lines were significantly shorter than those of the WT plants following independent inoculations with the *Xoo* strains GD1358, PXO347, and PXO340 (Fig. 6a–f). However, there were no significant differences in the LLs between the *OsSGT1*-*OE* rice lines and the WT plants following inoculation with JS97–2 (Fig. 6g, h). Thus, *OsSGT1* appears to positively regulate *Xoo* strain-specific resistance in rice.

### Transcriptome Profiling of the Defense Responses to Salt Stress and *Xoo* Infection Mediated by OsSAPK9

**Fig. 7:**
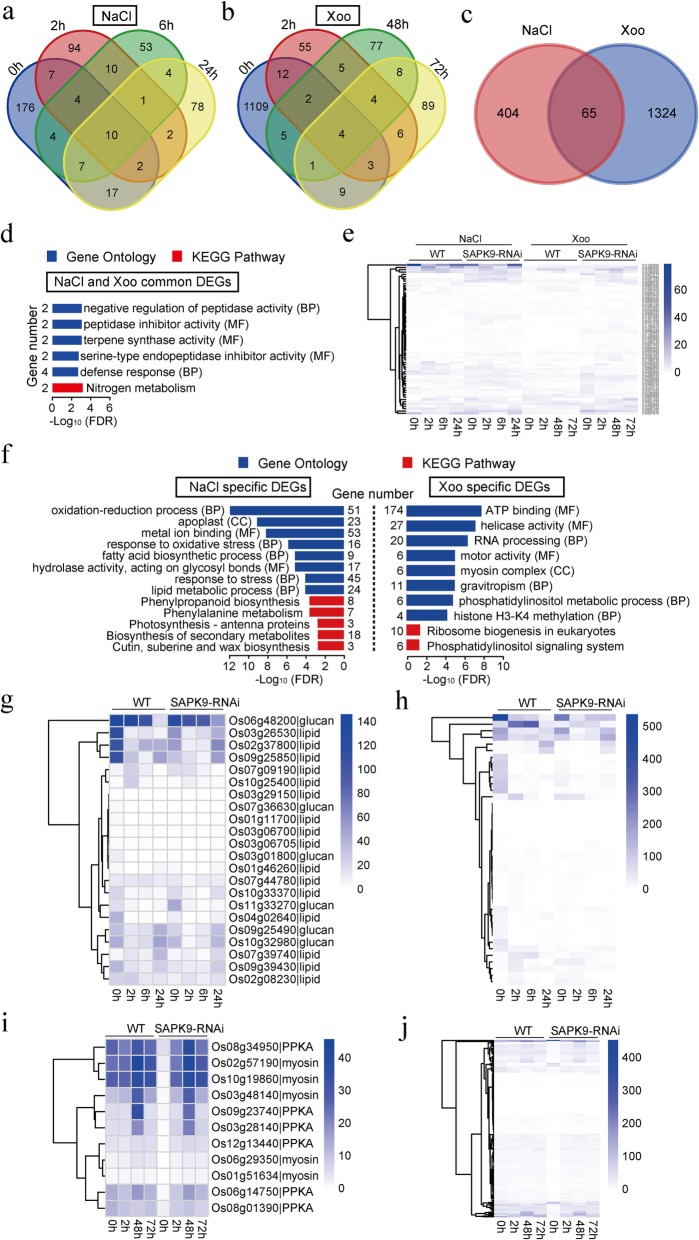
Differentially expressed genes (DEGs) regulated by *OsSAPK9* under salt-stress conditions and after inoculation with *Xanthomonas oryzae* pv. *oryzae* (*Xoo*), respectively. **a** Venn diagram showing the distribution of DEGs in wild-type (WT) vs *OsSAPK9-RNAi* plants under salt-stress conditions. **b** Venn diagram showing the distribution of DEGs in WT vs *OsSAPK9-RNAi* plants after inoculation with *Xoo.* **c** Venn diagram showing the distribution of DEGs in WT vs *OsSAPK9-RNAi* plants under salt-stress conditions and after inoculation with *Xoo.* **d** GO and KEGG terms of the 65 common DEGs identified under salt-stress conditions and after inoculation with *Xoo.* **e** Hierarchical clustering of the 65 common DEGs under salt-stress conditions and after inoculation with *Xoo.* **f** GO and KEGG terms of specific DEGs under salt-stress conditions and after inoculation with *Xoo*, respectively. **g** Hierarchical clustering of DEGs in the lipid and glucan metabolic pathways that are regulated by *OsSAPK9* under salt-stress conditions. **h** Hierarchical clustering of DEGs in the dynamics of the oxidation–reduction pathway that are regulated by *OsSAPK9* under salt-stress conditions. **i** Hierarchical clustering of DEGs in the phosphatidylinositol phosphate kinase activity and the cell cytoskeleton biosynthesis pathway that are regulated by *OsSAPK9* after inoculation with *Xoo*. PPKA, phosphatidylinositol phosphate kinase activity. **j** Hierarchical clustering of DEGs in the ATP energy signaling pathway that are regulated by *OsSAPK9* after inoculation with *Xoo*

To identify the responses mediated by *OsSAPK9*, the transcriptome profiles of *OsSAPK9-RNAi* line Ri-27 and WT plants were compared after exposure to salt stress and inoculation with the *Xoo* strain GD1358 (Additional file 10: Table S3). Using the criterion of *p* < 0.05, 469 differentially expressed genes (DEGs) were identified under salt-stress conditions when the WT was compared with *OsSAPK9*-*RNAi*. These included 130, 93, and 121 DEGs at 2 h, 6 h, and 24 h, respectively (Fig. 7a; Additional file 11: Table S4). When the plants were inoculated with GD1358, 1389 DEGs were identified in the comparison between WT and *OsSAPK9*-*RNAi*; including 91, 106, and 124 DEGs at 2 h, 48 h, and 72 h after inoculation, respectively (Fig. 7b; Additional file 12: Table S5). Among these, 65 DEGs that were significantly enriched in the Gene Ontology (GO) categories negative regulation of peptidase activity (*P* = 0.00128), peptidase inhibitor activity (*P* = 0.00128), terpene synthase activity (*P* = 0.00158), serine-type endopeptidase inhibitor activity (*P* = 0.00211), and defense response (*P* = 0.00321) were detected both under salt-stress conditions and after *Xoo* inoculation (Fig. 7c–e; Additional file 13: Table S6, Additional file 14: Table S7). Accordingly, there were 404 and 1324 DEGs that were unique to responses to the salt stress and *Xoo* infection, respectively (Fig. 7c). The DEGs specifically detected under salt-stress conditions were associated with oxidation–reduction process (*FDR* = 1.27E-12), response to oxidative stress (*FDR* = 1.10E-07), fatty acid biosynthetic process (*FDR* = 7.74E-06), and response to stress (*FDR* = 9.33E-05). (Fig. 7f; Additional file 13: Table S6, Additional file 14: Table S7). Glucan, fatty acids (FAs), and FA-derivatives are important sources of reserve energy and essential components of cell organelles in plants. They also play significant roles in improving stress tolerance in plants by participating in several defense-related pathways. The transcripts *LOC_Os03g01800*, *LOC_Os06g48200*, *LOC_Os07g36630*, *LOC_Os09g25490*, *LOC_Os10g32980*, and *LOC_Os11g33270* are involved in glucan metabolic processes and were differentially regulated in this study. *LOC_Os06g48200* and *LOC_Os11g33270* were down-regulated in the WT plants in comparison with *OsSAPK9*-*RNAi* plants, while the other transcripts were up-regulated in the same comparison (Fig. 7g; Additional file 15: Table S8). There were also 16 DEGs associated with lipid metabolic processes that were up-regulated in the WT plants (Fig. 7 g; Additional file 15: Table S8). Of these processes, redox regulation, antioxidant defense, and ROS signaling are critical in realizing and fine-tuning metabolic activities. There were 43 DEGs associated with the oxidation–reduction pathway, of which only 8 were down-regulated in the WT vs *OsSAPK9-RNAi* comparison (Fig. 7h; Additional file 15: Table S8). The majority of the 65 DEGs were up-regulated in the WT plants in comparison with the *OsSAPK9-RNAi* plants at 0 h, 2 h, and 6 h, but were down-regulated at 24 h. Thus, the activation of redox and metabolic signaling pathways may be delayed in *OsSAPK9-RNAi* plants in comparison with WT plants (Additional file 15: Table S8). DEGs induced by *Xoo* infection were associated with ATP binding (*FDR* = 1.52E-08), phosphatidylinositol metabolic process (*FDR* = 1.43E-05), and similar GO terms (Fig. 7f, Additional file 13: Tables S6, Additional file 14: Table S7). The phosphoinositide phosphate kinases are implicated in membrane trafficking and are important for plant growth, development, and the immune responses (Antignani et al. 2015; Gerth et al. 2017; Gou et al. 2015; Hempel et al. 2017). Six DEGs (*LOC_Os03g28140*, *LOC_Os06g14750*, *LOC_Os08g01390*, *LOC_Os08g34950*, *LOC_Os09g23740*, and *LOC_Os12g13440*) were enriched in the phosphatidylinositol phosphate kinase activity category, and all of these were down-regulated in the *OsSAPK9*-*RNAi* plants at 0 h and following *Xoo* inoculation (Fig. 7i; Additional file 16: Table S9). Five DEGs associated with the components of the myosin complex that maintains the stability of the cytoskeleton and cell movement (*LOC_Os02g57190*, *LOC_Os06g29350*, *LOC_Os03g48140*, *LOC_Os01g51634*, and *LOC_Os10g19860*) were down-regulated in the *OsSAPK9*-*RNAi* plants in comparison with the WT plants at 0 h, and these DEGs were up-regulated in the *OsSAPK9*-*RNAi* plants after inoculation with *Xoo* (Fig. 7i; Additional file 16: Table S9). In total, 19 of the 186 DEGs associated with the ATP-binding process were down-regulated in the WT plants in comparison with the *OsSAPK9*-*RNAi* plants, while the remainder were up-regulated. Even though most of the 186 DEGs were up-regulated in the *OsSAPK9*-*RNAi* plants after inoculation with *Xoo*, their expression did not significantly change compared with the WT plants inoculated with *Xoo* (Fig. 7j; Additional file 16: Table S9). Thus, the data suggest that the basic life signaling pathways, such as the ATP-binding process, cytoskeleton complex, and phosphatidylinositol phosphate kinase activity, are more rapidly down-regulated in *OsSAPK9*-*RNAi* plants than in WT plants after *Xoo* inoculation.

## Discussion

Plants have evolved cooperative and alternative molecular mechanisms to adapt to adverse environmental conditions. Protein kinases play central roles in signal recognition and the subsequent activation of plant defense mechanisms during pathogen infection. The SnRK2s are plant-specific and highly conserved protein kinases that affect responses to various stresses (Fujii et al. 2011; Kulik et al. 2011). In this study, we found that *OsSAPK9*, which belongs to the *SnRK2* gene family, positively regulates salt-stress tolerance and strain-specific resistance to bacterial blight in rice. According to our RNA-seq data, *OsSAPK9* regulated the downstream lipid and glucan metabolic pathways in plants after NaCl treatments, while it mainly regulated the ATP and cell cytoskeleton signaling pathways in plants inoculated with *Xoo* (Additional file 7: Figure S7). These findings demonstrate the potential of *OsSAPK9* as a tool for future crop improvements that may provide dual tolerances to salt and blight stresses. How is O*sSAPK9* involved in plant responses to the different stresses associated with saline conditions and pathogen infection? We found that *OsSGT1* might play a role in branch regulation by *OsSAPK9* in response to abiotic and biotic stresses in rice. The highly conserved eukaryotic co-chaperone SGT1 (a suppressor of the *G2* allele of *skp1*) is, in several plant species, a critical protein component of pattern- and effector-triggered immune responses against pathogens (Austin et al. 2002; Azevedo et al. 2002; Hoser et al. 2013; Shi et al. 2015; Tör et al. 2002). In rice, the overexpression of *OsSGT1* significantly increases strain-specific basal resistance to *Xoo* (Wang et al. 2008). Interactions between Hsp90 and SGT1 are required for the accumulation of resistance proteins and for the induction of disease resistance in a wide range of species (Ito et al. 2015; Kadota et al. 2008; Wang et al. 2015). In this study, we confirmed that OsSAPK9 forms a protein complex with OsSGT1 and the molecular chaperone OsHsp90 both in vivo and in vitro. Notably, *OsSGT1* positively regulated *Xoo* strain-specific resistance and negatively regulated the responses to salt stress (Figs. 5 and 6). This finding suggests that OsSGT1 plays a key role in diversifying the function of OsSAPK9 to activate different signaling pathways in response to abiotic and biotic stresses. We speculate that OsSAPK9 positively regulates rice resistance to *Xoo* through its interactions with OsSGT1 and OsHsp90. However, the mechanism by which OsSGT1 acts in the signaling pathway mediated by OsSAPK9 in response to salt stress is still obscure. Future research will focus on understanding the genetic relationship between OsSAPK9 and OsSGT1 to further characterize the molecular mechanism through which OsSAPK9 acts in response to diverse stresses.

## Conclusion

In this study, we revealed that *OsSAPK9* positively regulates salt-stress tolerance and bacterial blight resistance using *OsSAPK9-RNAi* and *OsSAPK9-overexpression* transgenic lines. RNA-seq data indicated that under salt-stress conditions and after *Xoo* inoculation, *OsSAPK9* not only regulates the respective specific signaling pathways but also regulates 65 common DEGs involved mainly in defense responses under both treatment conditions. We also revealed that OsSAPK9 interacts with OsSGT1 to regulate rice resistance to bacterial blight and salt stress. These results provide new insights into the mechanisms underlying the *OsSAPK9*-regulated resistance to biotic and abiotic stresses in rice.

## Materials and Methods

### Vector Construction and Rice Transformation

*OsSAPK9-RNAi* plants were created using a previously described RNAi strategy (Qiu et al. 2005). A 553-bp *OsSAPK9* cDNA fragment was amplified using PrimeSTAR GXL DNA Polymerase (TaKaRa, Dalian, China) and *O. sativa* ssp. *japonica* rice variety 9804 cDNA as the template (primers listed in Additional file 10: Table S2). The primers were specific to the 5′ and 3′ ends of the *OsSAPK9* fragment, and included the attB1 and attB2 adaptors, respectively. The resulting amplicon was cloned into the binary vector *pH7GWIWG2(II)*. To generate the *OsSAPK9-OE* and *OsSGT1*-*OE* lines, the full-length *OsSAPK9* and *OsSGT1* sequences, respectively, were downloaded from the Gramene database (http://www.gramene.org/) and amplified using PCR with the primers listed in Additional file 9: Table S2. The purified amplification products were independently cloned into the binary vector *pMDC43* for the subsequent production of a GFP-tagged fusion proteins. Gene expression was under the control of the Cauliflower mosaic virus 35S promoter. Rice varieties 9804 and Nipponbare were used to generate the transgenic plants. The above constructs were introduced into *Agrobacterium tumefaciens* strain EHA105 and incorporated into the genomes of 9804 and Nipponbare plants using an *A. tumefaciens*-mediated transformation method to generate the *OsSAPK9*-*RNAi* (Ri), *OsSAPK9-OE* (OE), and *OsSGT1-OE* (SA) lines. Ri-21 and Ri-27 are homozygous T_3_ RNAi transgenic lines, and OE1, OE2, SA24, and SA28 are T_2_ transgenic lines.

### PCR and Quantitative Real-Time PCR (qRT-PCR)

Putative transgenic lines were analyzed by PCR using hygromycin-specific primers (Additional file 9: Table S2). Genomic DNA was isolated from rice leaf samples using cetyl-trimethylammonium bromide as previously described (Saghai-Maroof et al. 1984). Total RNA was extracted from frozen samples using an RNAprep Pure Plant Kit (Tiangen, Beijing, China). The qRT-PCR assays were conducted using a TransScript Two-Step RT-PCR SuperMix (Trans, Beijing, China) with the primers listed in Additional file 9: Table S2. The rice *ACTIN2/8* gene was used as an internal control. The qRT-PCR analysis was conducted in triplicate, and the means of three biological replicates were used to represent the gene expression levels.

### Southern Blot Analysis

To estimate the number of copies of T-DNA fragments in the Ri-21 and Ri-27 lines, 15 μg genomic DNA was digested with *Hind*III restriction endonuclease, which did not cut within the T-DNA fragment. The digested DNA was fractionated on a 0.8% (w/v) agarose gel, blotted onto nylon membranes, and cross-linked (Sabelli 2007). The membranes were then hybridized with a 768-bp specific digoxigenin-labeled *Hyg* DNA fragment (Additional file 9: Table S2). Probe labeling and hybridization were conducted using a Digoxigenin-High Prime DNA Labeling and Detection Starter Kit II (Roche, Basel, Switzerland).

### Protein Extraction and Immunoblot Analysis

Proteins were extracted from *OsSAPK9-OE* and *OsSGT1*-*OE* rice seedlings and stored at − 80 °C. Protein extract concentrations were determined using a Bio-Rad Protein Assay Kit (Bio-Rad, CA, USA) with bovine serum albumin as the standard. Protein samples were separated electrophoretically on a 10% polyacrylamide gel containing protein markers. The proteins were subsequently transferred to a polyvinylidene fluoride membrane (Amersham, London, England) by semi-dry electroblotting (Mini-Protean II system; Bio-Rad). The membrane was blocked with 5% skim milk and blotted with a commercial GFP-tagged mouse monoclonal antibody (Abmart, Shanghai, China). After extensive washings, the bound primary antibody was detected with horseradish peroxidase-conjugated goat anti-mouse IgG secondary antibody according to the manufacturer’s recommendations (Abmart, Shanghai, China). The western blot experiment was repeated at least three times, with essentially the same results.

### Phenotypes, MDA Contents, and POD and CAT Activities of the Transgenic Rice Lines Under Salt-Stress Conditions

For the salt treatments, *OsSAPK9-RNAi* lines Ri-21 and Ri-27, *OsSAPK9-OE* lines OE1 and OE2, and *OsSGT1-OE* lines SA24 and SA28, and WT were used in this study. The seeds were surface-sterilized in 75% (v/v) ethanol for 2 min, 30% sodium hypochlorite for 30 min, and washed with distilled water for five times, then sowed on 1/2 MS medium (with and without hygromycin) at 25 °C under 16 h light/8 h dark cycles for 3 days. Then, the seedlings of transgenic lines and WT were cultured with Hoagland’s solution (Yoshida et al. 1976) in the greenhouse until two-leaf stages, 20-d-old seedlings were transferred to Hoagland’s solution supplemented with 100 mM NaCl, and the seedling survival rate was assessed daily. Additionally, 20-d-old seedlings were incubated in Hoagland’s solution supplemented with NaCl for 6 d, and fresh leaves (0.5 g) were harvested from and used to measure the MDA content and both the POD and CAT activity levels according to the methods described by Alkhateeb et al. (2015) and Shin et al. (2012), respectively.

### Artificial inoculation of WT and Transgenic Rice Lines with *Xoo*

To evaluate bacterial blight resistance, rice plants were cultivated in a screened house during the natural growing season. Seeds of WT, Ri-21, and Ri-27 plants were sown in a seedling nursery and 30-d-old seedlings were transferred to the screened house at the Institute of Crop Sciences, Chinese Academy of Agricultural Sciences, Beijing, China. Each line was planted in a single-row plot with 20 plants in each row (20 × 17 cm). Three replicates were used for each line. The transgenic plants (OE1, OE2, SA24, and SA28) were planted in a glasshouse at the Yunnan Academy of Agricultural Sciences, Yunnan, China. At the tillering stage (35 d after transplantation), 4–5 of the uppermost leaves of the plants in a row plot were inoculated with *Xoo* strains JS97–2, GD1358, PXO340, and PXO347 using the leaf-clipping method (Kauffman et al. 1973). The bacterial isolates were grown on peptone sucrose agar at 30 °C for 2 d, and the inocula were prepared by suspending the bacteria in sterile water to a final concentration of 10^8^ cells mL^− 1^. The LLs were measured on all the inoculated leaves 2 weeks after inoculation, which was when the lesions were most obvious and stable. The resistance level of each line was determined using the average LLs of five inoculated plants. Growth curves of the *Xoo* strain GD1358 in WT, Ri-21, Ri-27, OE1, OE2, SA24, and SA28 plants were produced using the method described by Song et al. (1995). Briefly, more than three leaf fragments per plant were sampled from the plants of all three transgenic lines after inoculation with *Xoo*. The surfaces of the leaf fragments were sterilized by immersion in 75% ethanol for 2 min, and then, the leaf fragments were cut and ground in a mortar with 1 mL sterilized water. The homogenate was diluted to the appropriate volume and 100 μL diluted homogenate from each of the samples was spread on PSA solid media. Finally, samples were incubated at 30 °C for 2 d, and then, the numbers of bacteria on three serial dilution plates that had measurable colony formation were counted.

### Plasmid Construction for Protein Expression

The CDS encoding 361 OsSAPK9 amino acids was amplified using the primers listed in Additional file 10: Table S2. The PCR products were inserted into the *pCold-TF* vector to express the histidine-tagged OsSAPK9 protein (His-OsSAPK9) in *Escherichia coli* (BL21) cells. The CDS encoding 367 OsSGT1 amino acids was amplified as well as the CDSs for the TPR (194 amino acids), CS (172 amino acids), and SGS (113 amino acids) domains using the primers listed in Additional file 9: Table S2. The sequences encoding a 275-amino acid fragment with ΔSGS and a 260-amino acid segment with ΔTPR were also amplified. The PCR products were cloned into the *pGEX-4T-1* vector using an In-Fusion Advantage PCR Cloning Kit (Clontech, Dalian, China) to generate plasmids for the production of the following fusion proteins in *E. coli* (BL21) cells: GST-OsSGT1, GST-TPR, and GST-CS. The *OsHsp90* CDS was also inserted into the *pGEX-4T-1* vector for the production of GST-OsHsp90 proteins.

### Yeast Two-Hybrid Assay

The vectors and yeast strains used in the yeast two-hybrid assays were obtained from Clontech. RNA was extracted at specific time points from leaves infected with *Xoo* strain PXO99A (1–5 d after infection). RNAs were mixed in equal proportions to construct an AD fusion cDNA library using the Matchmaker System with SMART cDNA synthesis technology (Clontech, Dalian, China). To further verify the interactions between OsSAPK9 and OsSGT1, the corresponding CDSs were amplified using gene-specific primer sets (Additional file 9: Table S2). The full-length *OsSAPK9* sequence was cloned into *pGBKT7* for the production of BD-OsSAPK9, while the full-length rice *OsSGT1* sequence was cloned into *pGADT7* to produce AD-OsSGT1, respectively. Yeast transformation, screening for positive clones, and subsequent reporter gene assays were carried out in accordance with the manufacturer’s instructions.

### Pull-Down and BiFC Assays

For the in vitro pull-down assays, full-length *OsSAPK9* sequence was inserted into the *pCold-TF* vector to produce His-OsSAPK9, and the full-length *OsSGT1* and *OsHsp90* sequences were inserted into the *pGEX4T-1* vector to produce GST-OsSGT1 and GST-OsHsp90, respectively (Additional file 9: Table S2). The fusion proteins and empty tags (GST or His) were produced in *E. coli* (BL21) cells and purified using the appropriate resin (Ni^2+^ for His tags and GST-binding resin for GST tags). GST-, GST-OsSGT1-, and GST-OsHsp90-coupled beads were used to capture the His tag or His-OsSAPK9. The pull-down assays were completed as described previously (Miernyk and Thelen 2008), and the proteins were detected with horseradish peroxidase-conjugated anti-His monoclonal antibodies (1:1000; Abmart). For the BiFC assay, *OsSAPK9* was cloned into the binary BiFC vector *pNYFP* (for N-terminal yellow fluorescent protein fusions) to produce *NYFP-OsSAPK9*, and *OsSGT1* and *OsHsp90* were cloned into the binary BiFC vector *pCCFP* (for C-terminal cyan fluorescent protein fusions) to produce *CCFP-OsSGT1* and *CCFP-OsHsp90*, respectively (Additional file 9: Table S2). These constructs, as well as the empty vectors, were introduced into *A. tumefaciens* strain EHA105 for the subsequent infiltration of 5-week-old *N. benthamiana* leaves as described by Huang et al. (2018). The leaves were observed 48–72 h after infiltration using an LSM 700 laser confocal scanning microscope (ZEISS Microsystems, Jena, Germany). The peak excitation wavelengths of YFP was 528 nm.

### Co-IP Assay

For the Co-IP assay, the *OsSAPK9* and *OsSGT1* CDSs were introduced into the binary vectors *pMDC43* (containing the GFP tag) and *pGWB18* (containing the Myc tag), respectively. These constructs were inserted into *A. tumefaciens* strain EHA105 for the infiltration of 5-week-old *N. benthamiana* leaves as described by Huang et al. (2018). Proteins were extracted from infiltrated leaves 48–72 h after infiltration. Samples were ground in liquid nitrogen, and proteins were extracted in Co-IP buffer (50 mM Tris-Cl, pH 7.4, 500 mM NaCl, 10% glycerol, 5 mM EDTA, 1% Triton X-100, and 1% Nonidet P-40) containing a protease inhibitor cocktail (Sigma-Aldrich, St. Louis, MO, USA). We used 10% of the extract as the input control. An agarose-conjugated Myc-tagged mouse monoclonal antibody (Abmart) solution was added to the extract, which was then incubated for 2 h at 4 °C. Beads were washed five times with Co-IP buffer lacking protease inhibitors. Sodium dodecyl sulfate (SDS)-polyacrylamide gel electrophoresis (PAGE) loading buffer (100 mM Tris-Cl, pH 6.8, 4% SDS, 0.2% bromophenol blue, 20% glycerol, and 200 mM dithiothreitol) was added to the eluted protein samples, which were then boiled for 5 min. We used 25% of the eluted proteins as the immunoprecipitation control. Anti-GFP and anti-Myc antibodies (Abmart) were used to detect the GFP-OsSAPK9 and MYC-OsSGT1 fusion proteins, respectively.

### RNA-seq of Transgenic and WT Lines

For the *OsSAPK9-RNAi-27* and WT lines treated with 100 mM NaCl or inoculated with *Xoo* strain GD1358, total RNA was extracted from the rice seedlings grown under 100 mM NaCl conditions and from 3-cm long seedling leaf tips of the rice seedling inoculated with GD1358 using TRIzol reagent according to the manufacturer’s instructions (Invitrogen, Waltham, MA, USA). For each sequencing library, 100 mg of RNA from each replicate was mixed together. The raw sequence data reported in this paper have been deposited in the Genome Sequence Archive (GSA) in the Beijing Institute of Genomics Data Center, Beijing Institute of Genomics, Chinese Academy of Sciences, under accession number PRJCA000702 and are publicly accessible at http://bigd.big.ac.cn/gsa (Wang et al. 2017). The libraries were sequenced by the CapitalBio Corporation (Beijing, China) using an Illumina HiSeq 2000 Sequencing System. Low quality nucleotides (< Q20) were trimmed from raw sequences for each sample, and then pair-end reads with either or both ends of lengths <30 bp were removed using an in-house Perl script. Retained high quality reads were mapped to the Michigan State University Rice Genome Annotation Project database (ftp://ftp.plantbiology.msu.edu/pub/data/Eukaryotic_Projects/o_sativa/annotation_dbs/pseudomolecules/version_7.0) using Bowtie (Langmead et al. 2009; Ouyang et al. 2007). The Cuffdiff module was used to identify DEGs. Chi-square tests were used to identify genes showing statistically significant differences in their relative abundance levels (as reflected by the total counts of individual sequence reads) between two samples using the IDEG6 software and a threshold of *p* ≤ 0.001 (Romualdi et al. 2003; Vencio et al. 2003; Ye et al. 2006). Pathway and GO enrichment analyses of rice DEGs were conducted using EXPath 2.0 (Chien et al. 2015). Venn diagrams were constructed using online software (http://bioinformatics.psb.ugent.be/webtools/Venn/).

### Accession Numbers

OsSAPK9: LOC_Os12g39630; OsSGT1: LOC_Os01g43540.

## Additional Files

**Additional file 1: Figure S1.** *OsSAPK9* expression under salt-stress conditions and after inoculation with *Xanthomonas oryzae* pv. *oryzae* (*Xoo*).

**Additional file 2: Figure S2.** Molecular characterization of *OsSAPK9-RNAi* and *OsSAPK9-overexpression* (*OsSAPK9-OE*) transgenic plants.

**Additional file 3: Figure S3.** *OsSAPK9* knock-down increases rice sensitivity to 50 μM NaCl in growth assays.

**Additional file 4: Figure S4.** Phenotypic responses of *OsSAPK9-RNAi*, *OsSAPK9*-*overexpression* (*OsSAPK9-OE*), and wild-type (WT) plants inoculated with *Xanthomonas oryzae* pv. *oryzae* (*Xoo*).

**Additional file 5: Figure S5.** The Coomassie Brilliant blue staining results of a pull-down assay.

**Additional file 6: Figure S6.** Molecular characterization of *OsSGT1-overexpression* (*OsSGT1-OE*) transgenic plants.

**Additional file 7: Figure S7.** Model regulated by *OsSAPK9* under salt-stress conditions and after inoculation with *Xanthomonas oryzae* pv. *oryzae* (*Xoo*), respectively.

**Additional file 8: Table S1.** Yeast two-hybrid analysis of proteins interacting with OsSAPK9.

**Additional file 9: Table S2.** Details of the primers used in this study. (PDF 76 kb)

**Additional file 10: Table S3.** The summary of RNA-seq data under salt-stress conditions and after inoculation with *Xanthomonas oryzae* pv. *oryzae* (*Xoo*).

**Additional file 11: Table S4.** Differentially expressed genes (DEGs) in wild-type (WT) compared with the *OsSAPK9-RNAi* line, under salt-stress conditions.

**Additional file 12: Table S5.** Differentially expressed genes (DEGs) in wild-type (WT) compared with the *OsSAPK9-RNAi* line after inoculation with *Xanthomonas oryzae* pv. *oryzae* (*Xoo*).

**Additional file 13: Table S6.** GO terms of differentially expressed genes (DEGs) in wild-type (WT) compared with *OsSAPK9-RNAi* under salt-stress conditions and after inoculation with *Xanthomonas oryzae* pv. *oryzae* (*Xoo*).

**Additional file 14: Table S7.** KEGG pathways of differentially expressed genes (DEGs) in wild-type (WT) compared with *OsSAPK9-RNAi* under salt-stress conditions and after inoculation with *Xanthomonas oryzae* pv. *oryzae* (*Xoo*).

**Additional file 15: Table S8.** Differentially expressed genes (DEGs) associated with oxidation reduction, and glucan and lipid metabolic processes in rice treated with salt stress.

**Additional file 16: Table S9.** Differentially expressed genes (DEGs) associated with phosphatidylinositol phosphate kinase activity, myosin complex, and ATP-binding processes in rice inoculated with *Xanthomonas oryzae* pv. *oryzae* (*Xoo*).

## Data Availability

Not applicable.

## Abbreviations

CAT: Catalase
DEGs: Differentially expressed genes
LLs: Lesion lengths
MDA: Malondialdehyde
POD: Peroxidase
SAPK9: Stress-activated protein kinases 9
SnRK2: Sucrose non-fermenting 1 (SNF1)-related protein kinase 2
*Xoo*: *Xanthomonas oryzae* pv. *oryzae*
