# Supplementary figures and images for: Stress-Activated Protein Kinase OsSAPK9 Regulates Tolerance to Salt Stress and Resistance to Bacterial Blight in Rice
Source: Rice (N Y). 2019 Nov 11;12:80. doi: 10.1186/s12284-019-0338-2 (PMC6848426; doi:10.1186/s12284-019-0338-2)

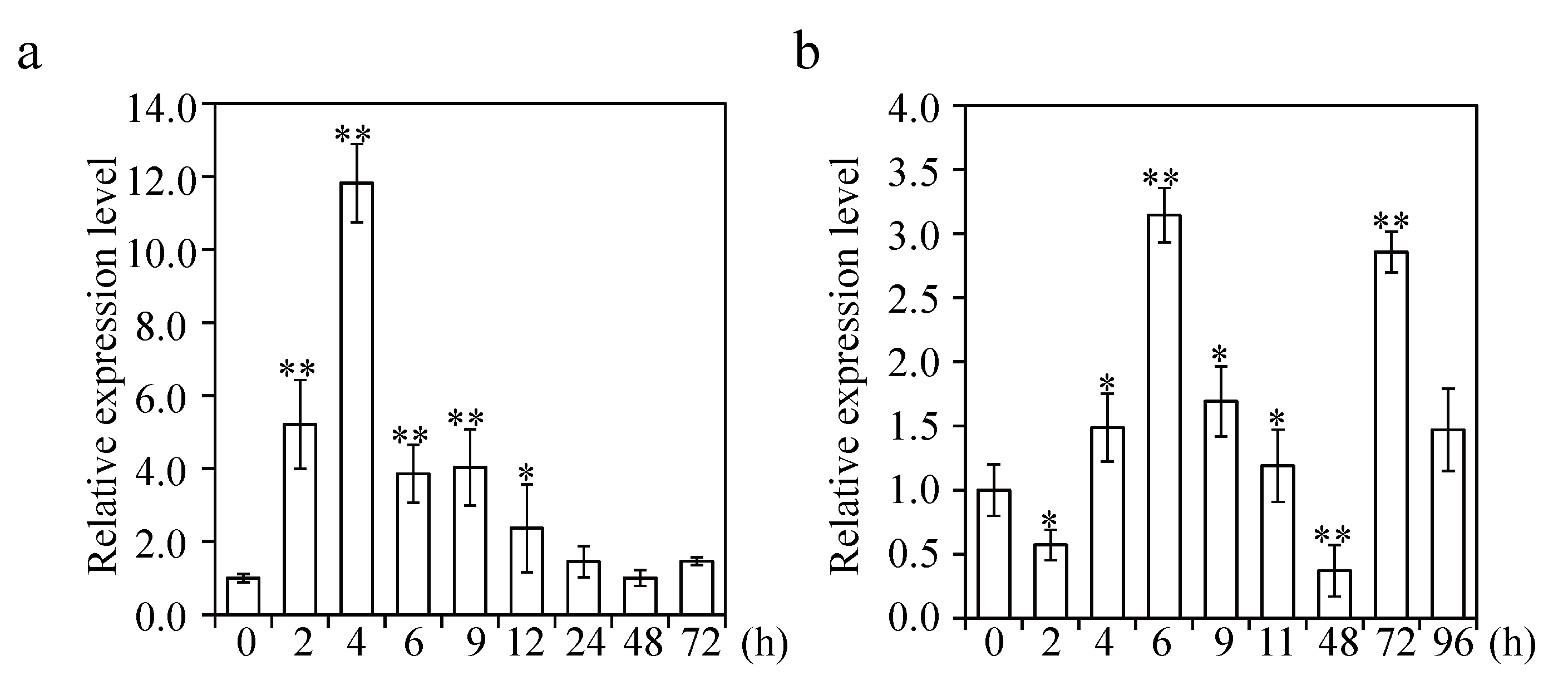

Supplement: Supplementary file 1 — Additional file 1: Figure S1. OsSAPK9 expression under salt-stress conditions and after inoculation with Xanthomonas oryzae pv. oryzae (Xoo). [file 12284_2019_338_MOESM1_ESM.tif]

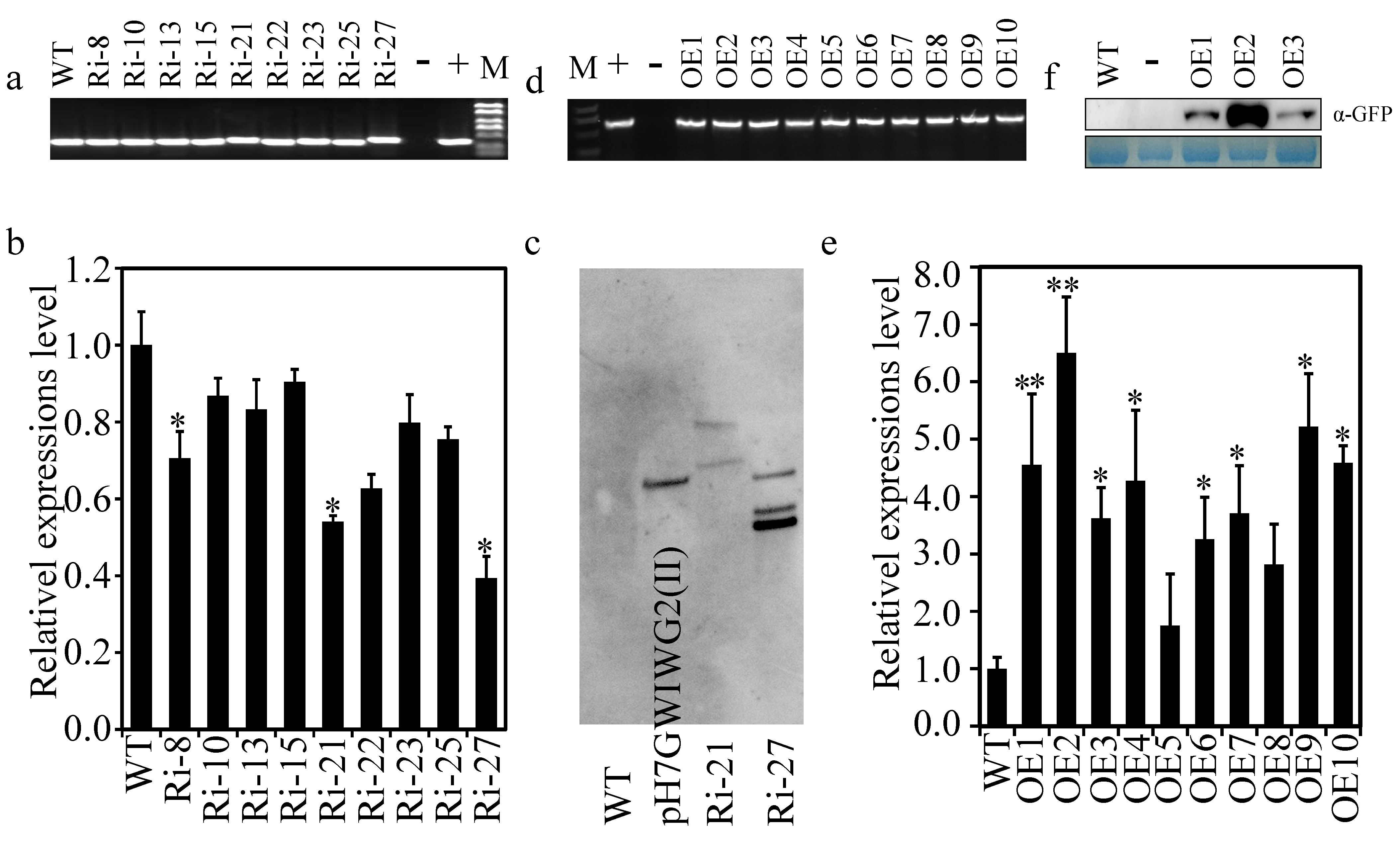

Supplement: Supplementary file 2 — Additional file 2: Figure S2. Molecular characterization of OsSAPK9-RNAi and OsSAPK9-overexpression (OsSAPK9-OE) transgenic plants. [file 12284_2019_338_MOESM2_ESM.tif]

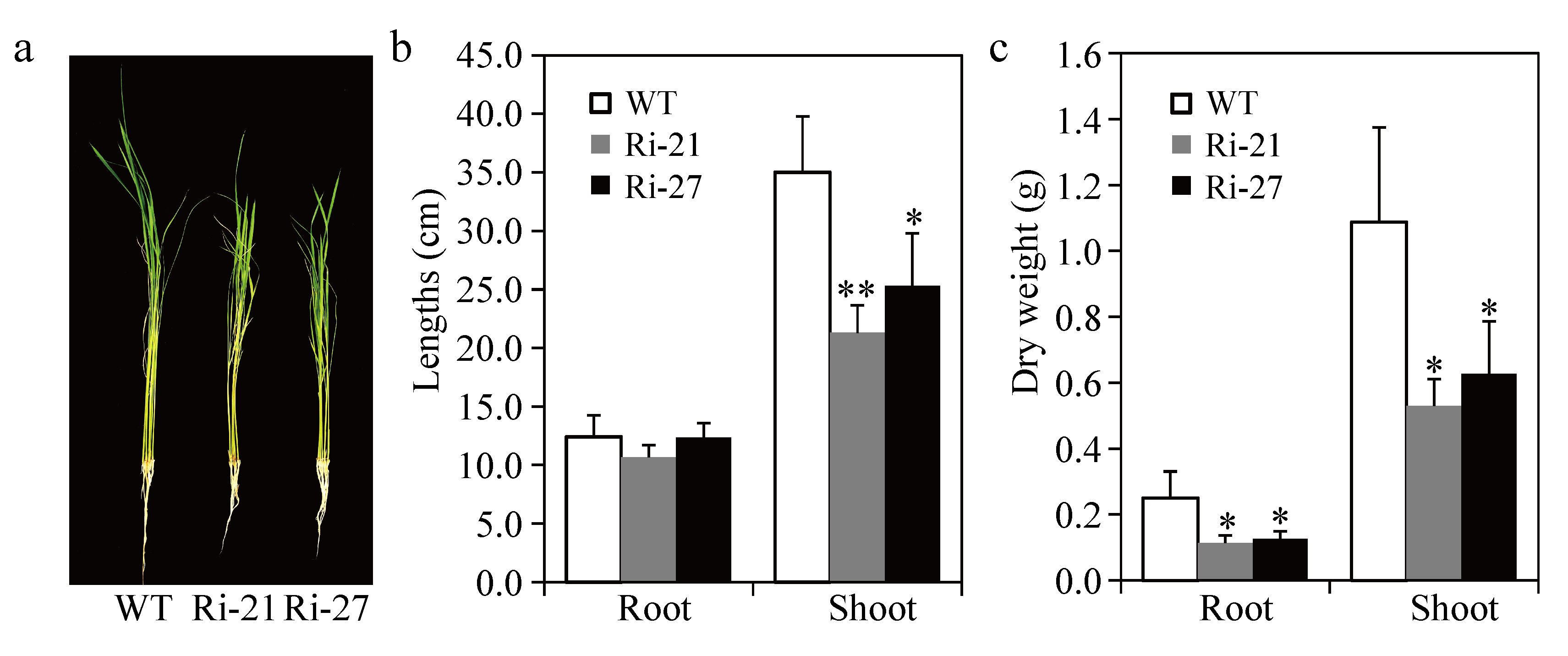

Supplement: Supplementary file 3 — Additional file 3: Figure S3. OsSAPK9 knock-down increases rice sensitivity to 50 μM NaCl in growth assays. [file 12284_2019_338_MOESM3_ESM.tif]

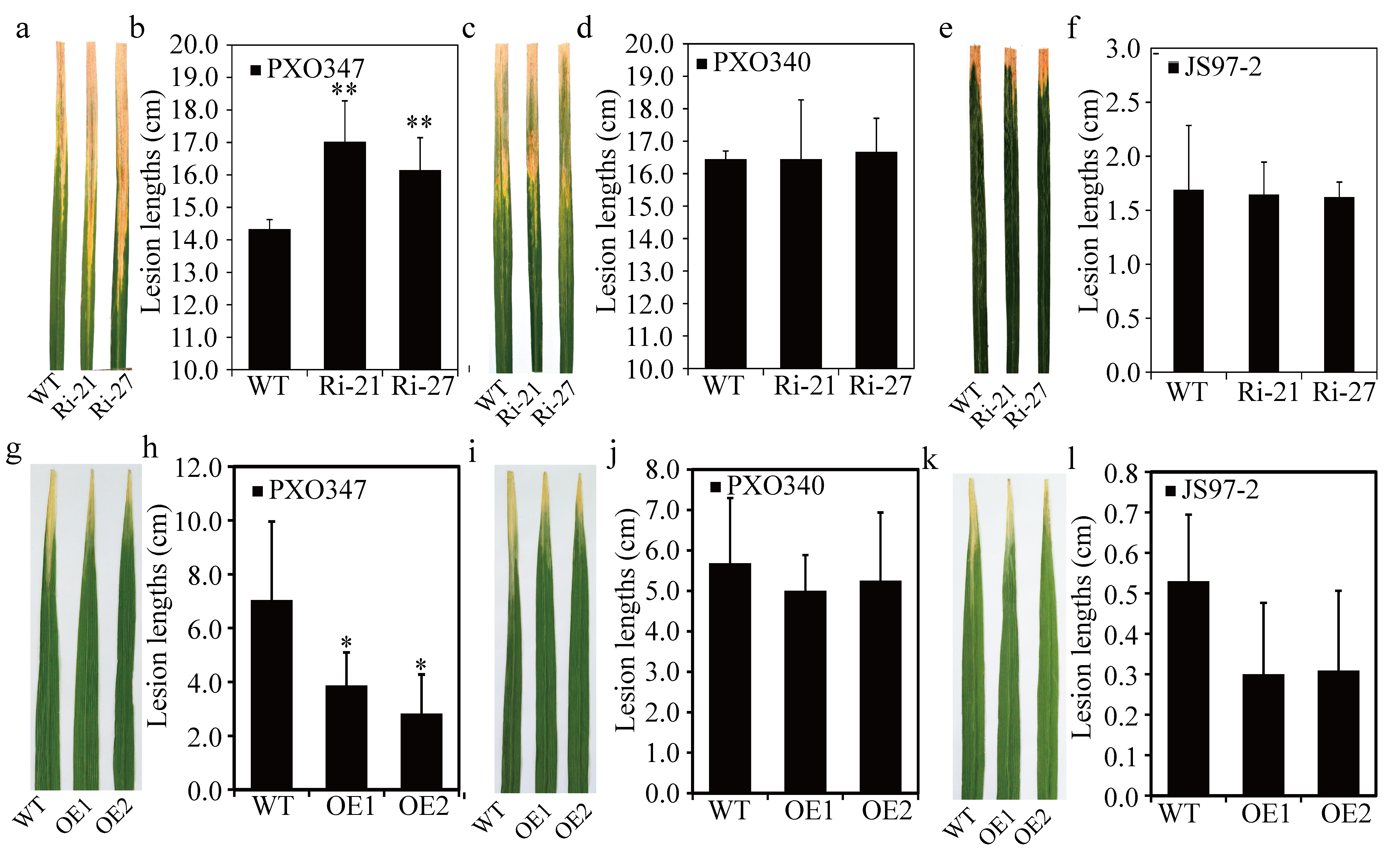

Supplement: Supplementary file 4 — Additional file 4: Figure S4. Phenotypic responses of OsSAPK9-RNAi, OsSAPK9-overexpression (OsSAPK9-OE), and wild-type (WT) plants inoculated with Xanthomonas oryzae pv. oryzae (Xoo). [file 12284_2019_338_MOESM4_ESM.tif]

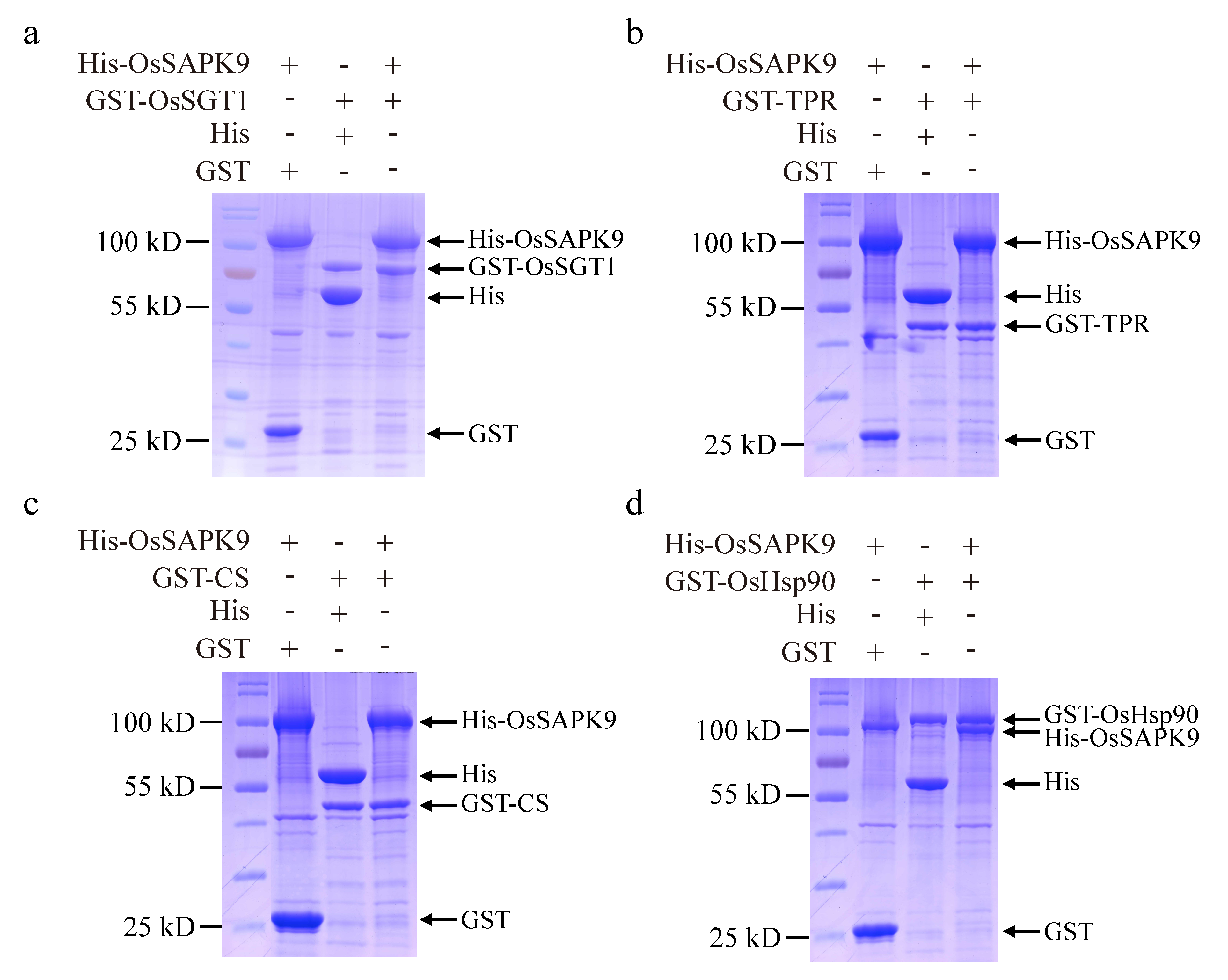

Supplement: Supplementary file 5 — Additional file 5: Figure S5. The Coomassie Brilliant blue staining results of a pull-down assay. [file 12284_2019_338_MOESM5_ESM.tif]

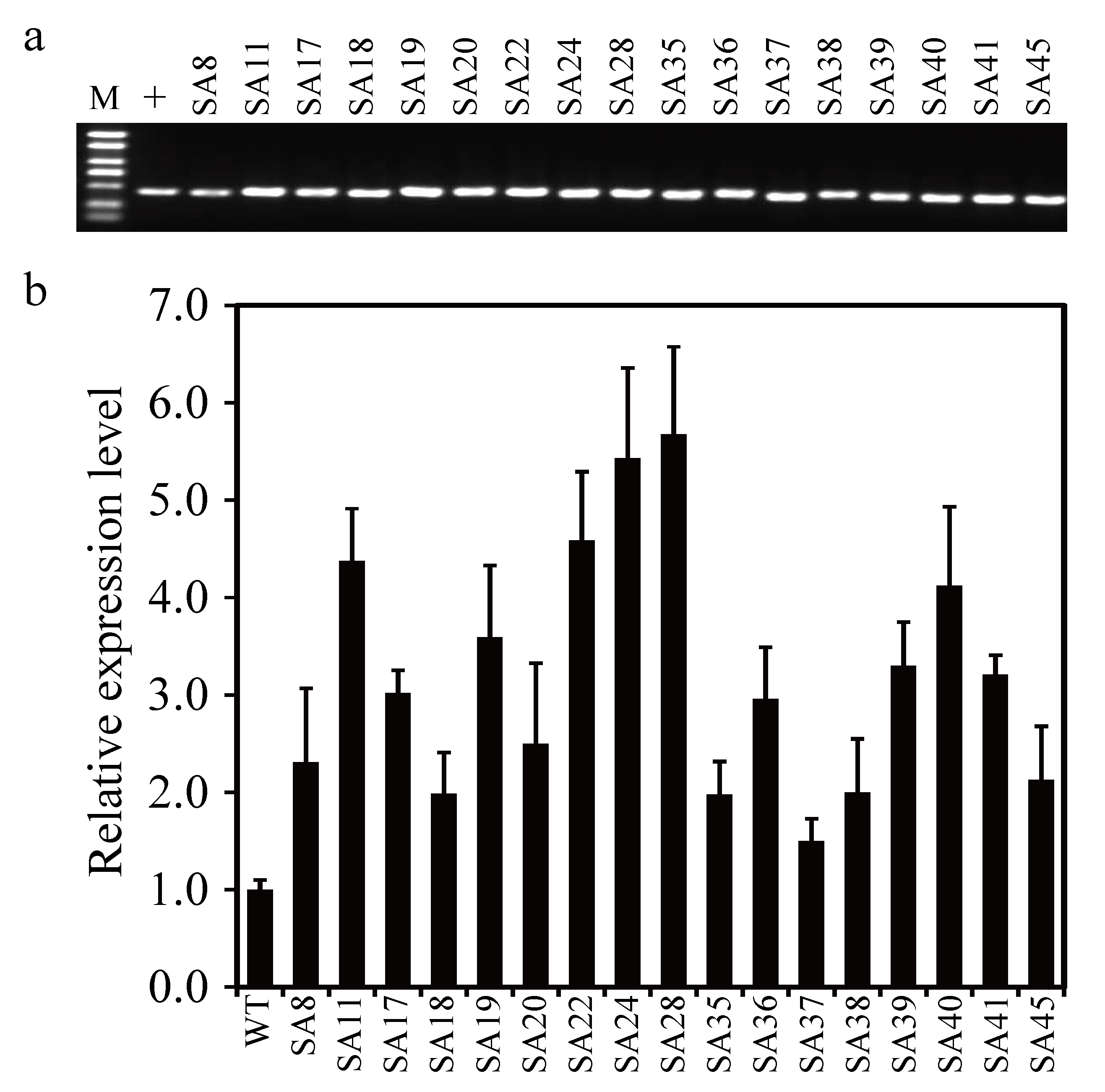

Supplement: Supplementary file 6 — Additional file 6: Figure S6. Molecular characterization of OsSGT1-overexpression (OsSGT1-OE) transgenic plants. [file 12284_2019_338_MOESM6_ESM.tif]

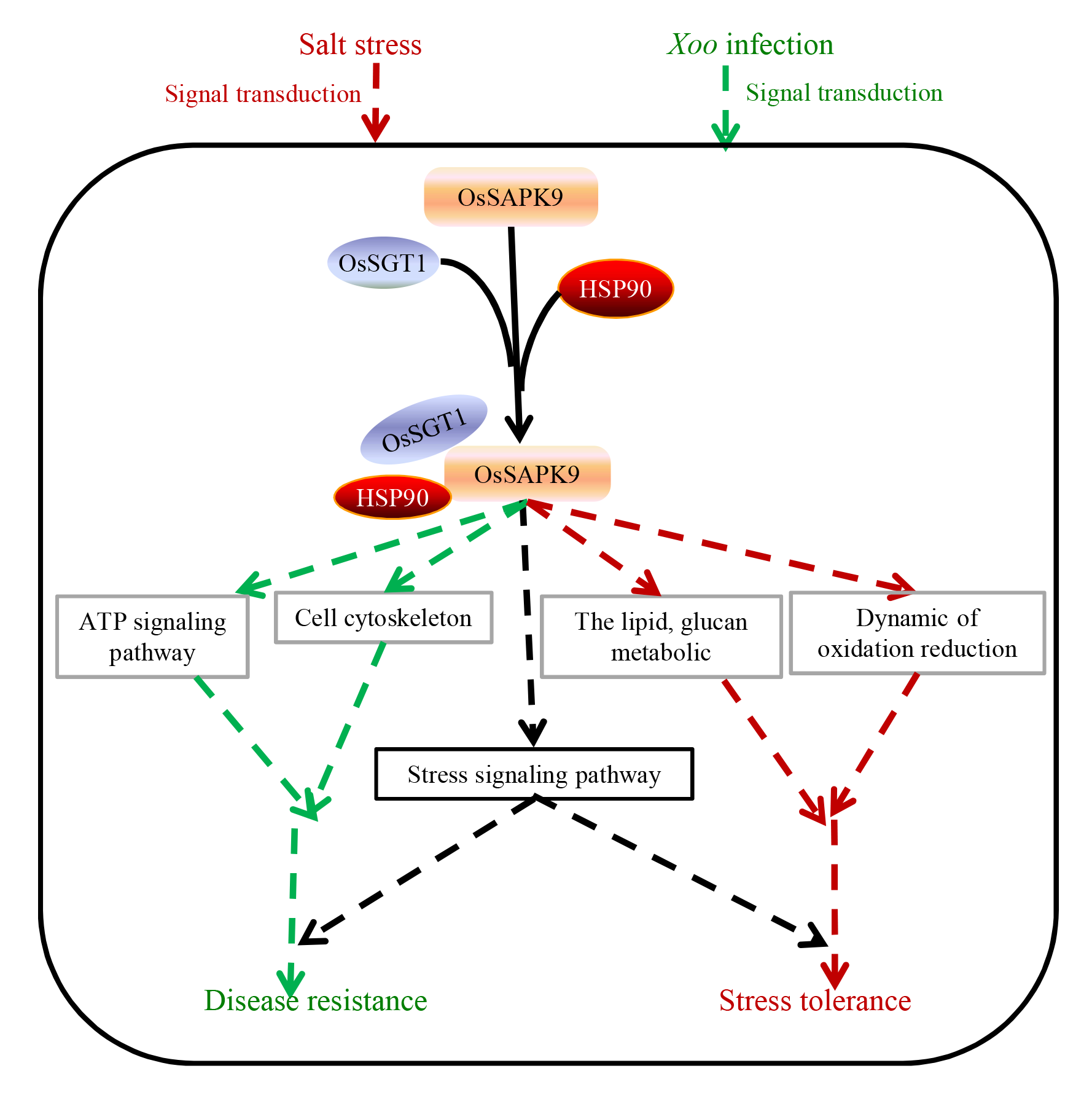

Supplement: Supplementary file 7 — Additional file 7: Figure S7. Model regulated by OsSAPK9 under salt-stress conditions and after inoculation with Xanthomonas oryzae pv. oryzae (Xoo), respectively. [file 12284_2019_338_MOESM7_ESM.tif]
