# Supplementary material for: Stress-Activated Protein Kinase OsSAPK9 Regulates Tolerance to Salt Stress and Resistance to Bacterial Blight in Rice
Source: Rice (N Y). 2019 Nov 11;12:80. doi: 10.1186/s12284-019-0338-2 (PMC6848426; doi:10.1186/s12284-019-0338-2)
Supplement: Supplementary file 8 — Additional file 8: Table S1. Yeast two-hybrid analysis of proteins interacting with OsSAPK9. [file 12284_2019_338_MOESM8_ESM.pdf]

Table S1 Interacting proteins analysis of OsSAPK9 by yeast two hybrid (Y2H).

Number	Gene_LOC	Annotion
S9-11	LOC_Os05g28980	Dethydration-induced 19 homolog 2 (OsDi19-2)
S9-12	LOC_Os01g01410	C-type lectin domain containing protein
S9-13	LOC_Os02g57280	OsCBSX3; CBS domain containing protein
S9-18	LOC_Os04g53994	S-locus receptor-like kinase RLK14； Similar to Receptor-like protein kinase 5
S9-22	LOC_Os10g30580	Similar to Cell division control protein 48 homolog A
S9-28	LOC_Os04g46390	Heat shock protein DnaJ family protein (hsp40)
**S9-29**	**LOC_Os01g43540**	**OsSGT1**
S9-30	LOC_Os09g36220	pseudo-response regulator 95
S9-31	LOC_Os03g28940	ZIM domain containing protein; OsJA5
S9-34	LOC_Os03g13170	ubiquitin fusion protein; the ubiquitin protein fused to a ribosomal protein
S9-36	LOC_Os09g36220	pseudo-response regulator 95
S9-41	LOC_Os07g39300	Conserved hypothetical protein
S9-42	LOC_Os02g54150	Similar to SAC domain protein 1
S9-51	LOC_Os03g11410	Mitochondrial processing peptidase beta subunit
S9-80	LOC_Os07g42450	Similar to 40S ribosomal protein SA (p40)
S9-92	LOC_Os09g33710	Full=Beta-glucosidase 32
S9-103	LOC_Os05g47980	ATP synthase beta chain, mitochondrial precursor
S9-105	LOC_Os01g74460	Vacuolar fusion protein MON1 domain containing protein
S9-114	LOC_Os03g07880	CCAAT-binding transcription factor subunit B family protein
S9-125	LOC_Os09g39540	Similar to Ribosomal protein S25
S9-130	LOC_Os05g07130	Similar to Fructose-6-phosphate 2-kinase/fructose-2,6-bisphosphatase
S9-133	LOC_Os04g37619	Similar to Zeaxanthin epoxidase OsZEP-1
S9-139	LOC_Os01g44210	Similar to 50S ribosomal protein L31

S9-141	LOC_Os07g48490	Ricin B-related lectin domain containing protein
S9-171	LOC_Os07g37250	THYLAKOID FORMATION1, chloroplast precursor
S9-174	LOC_Os04g11400	Conserved hypothetical protein
S9-182	LOC_Os03g58590	Similar to Cactin
S9-189	LOC_Os04g42120	Similar to N-acetyltransferase ESCO1
S9-65	LOC_Os03g63950	Similar to Plastid-specific 30S ribosomal protein 1, chloroplast precursor
S9-69	LOC_Os05g51700	Nucleoside Diphosphate Kinase 3
